# Child type 1 diabetes associated with mother vaginal bacteriome and mycobiome

**DOI:** 10.1007/s00430-022-00741-w

**Published:** 2022-06-14

**Authors:** A. L. Ruotsalainen, M. V. Tejesvi, P. Vänni, M. Suokas, P. Tossavainen, A. M. Pirttilä, A. Talvensaari-Mattila, R. Nissi

**Affiliations:** 1 Department of Ecology and Genetics, University of Oulu, POB 3000, 90014 Oulu, Finland; 2 Department of Obstetrics and Gynecology, University of Oulu, PL 23, FI90029 Oulu, Finland; 3 Genobiomics LLC, Oulu, Finland; 4 Department of Pediatrics, PEDEGO Research Unit and Medical Research Center, University of Oulu and Oulu University Hospital, PO Box 23, 90029 OYS Oulu, Finland; 5 Biocenter Oulu Sequencing Center, University of Oulu, POB 8000, 90014 Oulu, Finland

**Keywords:** Machine learning, Microbial diversity, Next generation sequencing, Vaginal microbiome

## Abstract

Mother vaginal microbes contribute to microbiome of vaginally delivered neonates. Child microbiome can be associated with autoimmune diseases, such as type 1 diabetes (T1D). We collected vaginal DNA samples from 25 mothers with a vaginally delivered child diagnosed with T1D and samples from 24 control mothers who had vaginally delivered a healthy child and analyzed bacteriome and mycobiome of the samples. The total DNA of the samples was extracted, and ribosomal DNA regions (16S for bacteria, ITS2 for fungi) were amplified, followed by next-generation sequencing and machine learning. We found that alpha-diversity of bacteriome was increased (*P* < 0.002), whereas alpha-diversity of mycobiome was decreased (*P* < 0.001) in mothers with a diabetic child compared to the control mothers. Beta-diversity analysis suggested differences in mycobiomes between the mother groups (*P* = 0.001). Random forest models were able to effectively predict diabetes and control status of unknown samples (bacteria: 0.86 AUC, fungi: 0.96 AUC). Our data indicate several fungal genera and bacterial metabolic pathways of mother vaginal microbiome to be associated with child T1D. We suggest that early onset of T1D in a child has a relationship with altered mother vaginal microbiome and that both bacteriome and mycobiome contribute to this shift.

**Supplementary Information:**

The online version contains supplementary material available at 10.1007/s00430-022-00741-w.

## Introduction

Mother vaginal microbiome contributes to the microbial community (i.e., microbiome, including bacteria and fungi) of gut, oral cavities and skin of vaginally delivered children [1–3]. The child gut microbiome, on the other hand, is associated with autoimmune diseases, such as type 1 diabetes (T1D), during childhood or later in life [4]. Restoration of gut microbiota with *Bifidobacterium infantis* in early life could protect a child from development of T1D [5]. Children born by cesarean section (CS) have altered microbial communities in the gut compared to vaginally delivered children [1, 6, 7] and CS has been considered as a risk factor for the early onset of T1D [8]. Regardless of such associations, the connection between the vaginal microbiome and child microbiome in the development of T1D may be complex [9, 10]. In a case–control-type pilot study, Tejesvi et al. [11] found that the mother vaginal microbiome and child T1D may be associated. The results by Tejesvi et al. suggested that mothers who gave birth to a child with T1D had a more diverse vaginal microbiome than control mothers.

The full microbiome, which has concerned only bacterial species in the large majority of studies, but includes also human fungal communities, i.e., mycobiomes that were long neglected but have lately caught attention [12]. Specifically, the role of human mycobiome and association of the dysbiosis between bacteriome and mycobiome with autoimmune diseases has been under investigation [4, 13, 14]. Fungal and bacterial dysbiosis and intestinal inflammation of neonates was recently associated with beta-cell autoimmunity, as children who developed T1D had high amounts of *Saccharomyces* and *Candida* yeasts in their gut [4]. Vaginal mycobiome has been relatively little studied to date. However, studies by Drell et al. [15] and Bradford and Ravel [16] suggest that the vaginal mycobiome is more diverse than previously assumed. Drell et al. noted that the low quality of reference libraries and poor knowledge of fungal taxonomy may complicate the analyses. Nevertheless, interactions between bacterial and fungal communities, especially between *Lactobacillus* spp. and *Candida* spp., have been considered fundamental for future research [16, 17].

We compared bacteriome and mycobiome samples of mothers who had delivered at least one child diagnosed with T1D by the age of 11 (*N* = 25, group hereafter named as Diabetes group) with similar samples from control mothers with children without diabetes (*N* = 24, group hereafter named as Control group) using next-generation sequencing and machine learning approach. The aim of this study was (1) to find out whether association of mother vaginal microbiome and child T1D found by Tejesvi et al. [11] can be corroborated to a larger sampling from the same research population. We also aimed (2) to find out whether further differences in vaginal mycobiomes exist with respect to the mother groups (Diabetes group vs. Control group). Finally, (3) we analyzed the predicted metabolic pathways to find out whether child T1D-specific signatures were detectable in this study population.

## Methods

### Description of samples and sampling protocol

Material for this study was collected in the Northern Ostrobothnia Hospital District 2018–2020 covering population in Oulu area, Northern Finland. Institutional Ethics Committee approved the study and the patient consent (Statement of the regional Ethics committee 21.6.2017). All research was performed in accordance with relevant regulations, and informed consent was obtained from all participants. The material for the study was collected from 25 mothers with at least one child with T1D by vaginal delivery (child age less than 11 years at the time of sampling) and 24 control mothers with at least one vaginal delivery and no diagnosed child/children with diabetes. The age of the mothers was set to be between 22 and 40 years at the time of sampling. Mothers included in this study did not have hormonal birth control or constant medication. No other background data on mothers were collected in this study. The samples were collected in a normal protocol of vaginal surface sampling. Sampling was carried out by two specialist doctors with one mainly taking samples from Diabetes group and one taking samples from Control group, by following exactly same protocol in sampling. After sampling, the tip of the swab was immediately placed into a sterile Eppendorf tube and preserved at  – 20 °C. Data is deposited in GenBank https://www.ncbi.nlm.nih.gov/genbank/ under the bioproject numbers PRJNA751475 for bacteria https://dataview.ncbi.nlm.nih.gov/object/PRJNA751475?reviewer=jia44284elai976f8b5otp063r and PRJNA751714 for fungi https://dataview.ncbi.nlm.nih.gov/object/PRJNA751714?reviewer=h1oaitfif938522cmfv3r5frq

### PCR amplification, DNA extraction, and sequencing

DNeasy Power Soil® Pro DNA isolation kit (Qiagen) and Qiacube robotic workstation (Qiagen) were used to extract genomic DNA from frozen cotton swabs to identify bacterial and fungal communities. Before PCR amplification, genomic DNA was diluted to a concentration of 10 ng/µl and analyzed using a Nanodrop spectrophotometer.

The Primers 519F (5'-CAGCMGCCCGCGGTAATWC-3') and 926R (5'-CCGTCAATTCCTTTRAGTTT-3') were used to amplify a portion of the bacterial 16S small ribosomal unit gene in bacterial Polymerase chain reactions (PCR). The 519F primer had a 30-bp long adapter sequence A, a 9-bp specific barcode sequence for each sample, and a single nucleotide linker A at the beginning of the Ion Torrent sequencing method. The Ion Torrent adapter series trP1 was present at the beginning of the 926R primer. 1 × Phusion Flash High-Fidelity Master Mix (ThermoFisher Scientific), 0,5 M forward and reverse primers, and 10 ng template DNA were used in duplicate polymerase chain reactions in a 15 µl volume. After a 3-min denaturation period at 98 °C, the following conditions were used for 22 cycles: 98 °C, 10 s; 64 °C, 10 s; 72 °C, 30 s. The final extension was done for 5 min at 72 °C.

With primers ITS4 (5'-TCCTCCGCTTATTGATATGC-3') and fITS7 (5'- GTGARTCATCGAATCTTTG -3'), the ITS2 region of the ribosomal RNA gene was amplified for fungal community analysis. The ITS4 primer had a 30-bp long adapter sequence A and a 10-bp unique multiplex identifier sequence (MID) at the beginning, while the fITS7B primer had a 30-bp long adapter sequence A and a 10-bp unique multiplex identifier sequence (MID) at the beginning. PCR amplification was performed in the same manner as for 16S rRNA products, with an initial denaturation at 98 °C for 2 min, followed by 32 cycles of 10 s at 98 °C, 20 s at 54 °C, and 30 s at 72 °C, and a final extension for 7 min at 72 °C.

Both PCR reactions were done in triplicate, and the PCR products were analyzed on an agarose gel. Following that, the triplicate reactions were mixed, cleaned with a Beckman Coulter Agencourt AMPure XP PCR purification system, and quantified with an Agilent Bioanalyzer using the DNA-1000 analysis package (Agilent). Individual 16S and ITS samples were then pooled at equimolar ratios for sequencing, and the pools were purified with Ampure XP, tested for purity with a bioanalyzer, and concentration was measured with a picogreen assay. Sequencing was performed using Ion Torrent PGM sequencer, 316 v2 chip, Ion PGM Hi-Q View template kit (400 bp templating program) and Ion PGM Hi-Q View Sequencing kit (850 cycles).

### Bioinformatics: 16S sequence and ITS sequence preprocessing

*Multiplexed 16S sequences* were imported into Qiime2 (version 2019.10) [18]. Barcode sequences were removed using the q2-cutadapt-plugin [19]. Primer sequences (f-primer: CAGCMGCCGCGGTAATWC, r-primer: CCGTCAATTCCTTTRAGTTT) were removed using the q2-cutadapt-plugin. Sequences were denoised to ASV's using the q2-dada2-plugin [20] with truncation length parameter set to 391 base pairs. Naive-Bayes taxonomic classifier was trained using the SILVA (v138) database [21] trimmed to the forward and reverse primers used in sequencing and truncated to 391 bp length. Chimeric sequences were detected and removed using the q2-vsearch-plugin [22]. Features found only in one sample and those with less than 10 frequency across all samples were removed. Taxonomy was assigned using the naive-bayes classifier. Non-bacterial ASV's, mitochondria, and chloroplast sequences were removed using the q2-taxa-plugin. At this point, samples that had a total feature frequency lower than 1000 were removed. Additionally, the ASV-table was then collapsed into a taxonomic level of genera and the metabolic pathway composition was predicted using the q2-picrust2-plugin [23]. The q2-picrust–plugin outputted MetaCyc [24] metabolic pathways. *ITS sequences* were preprocessed similarly to 16S sequences, except 311 was chosen as the q2-dada2 truncation length, and the R-package Decontam [25] was used to identify and remove features identified as contaminants. Taxonomy was assigned to ITS features using the UNITE (v8.2) database [26].

### Diversity analyses and differential abundance

Alpha and beta diversity analyses were done using the q2-diversity-plugin and visualized with Matplotlib python package. Shannon index and Bray–Curtis dissimilarity were chosen as diversity metrices. ASV-tables were rarefied to the sampling depth of 1000, while metabolic pathway data were rarefied to a depth of 10,000. Principal coordinates analysis (PCoA) was performed using the q2-diversity-plugin with bacteria, predicted pathways and fungi data independently. Statistical differences between diabetes and control samples were tested with Kruskal–Wallis H-test with Scipy and PERMANOVA with q2-diversity-plugin in alpha and beta diversity, respectively. Statistical differences in comparison of individual taxonomic groups were carried out using Kruskal–Wallis H-test in R environment (version 4.1.0, [27]). Differentially abundant genera and predicted pathways were investigated using the q2-aldex2-plugin [28].

### Machine learning

Random forest [29] and logistic regression models were trained to predict the diabetes status of the samples using scikit-learn package [30]. Nested cross-validation scheme with tenfolds in each layer was used. In tenfold cross-validation, the whole data are first partitioned to ten different validation and training folds, where each sample is once in the validation fold. In nested cross-validation, a second tenfold cross-validation split is done on the training fold to tune optimal parameters. This way, the validation fold is unseen to the training process of the models. Default parameters were used for random forests, while parameter “C” was tuned using the training folds for logistic regression models. Feature importance values were gathered during model training, where random forest models outputted the normalized gini importance and logistic regression the feature coefficients. Area under the curve (AUC) of receiver operating characteristic (ROC) were chosen as the performance metric as it performs well with class unbalanced data. Nested cross-validation process was repeated 40 times and the model performances, feature importance’s and coefficient values from each cross-validation iteration were pooled together, averaged, and finally plotted using Matplotlib.

## Results

**Fig. 1:**
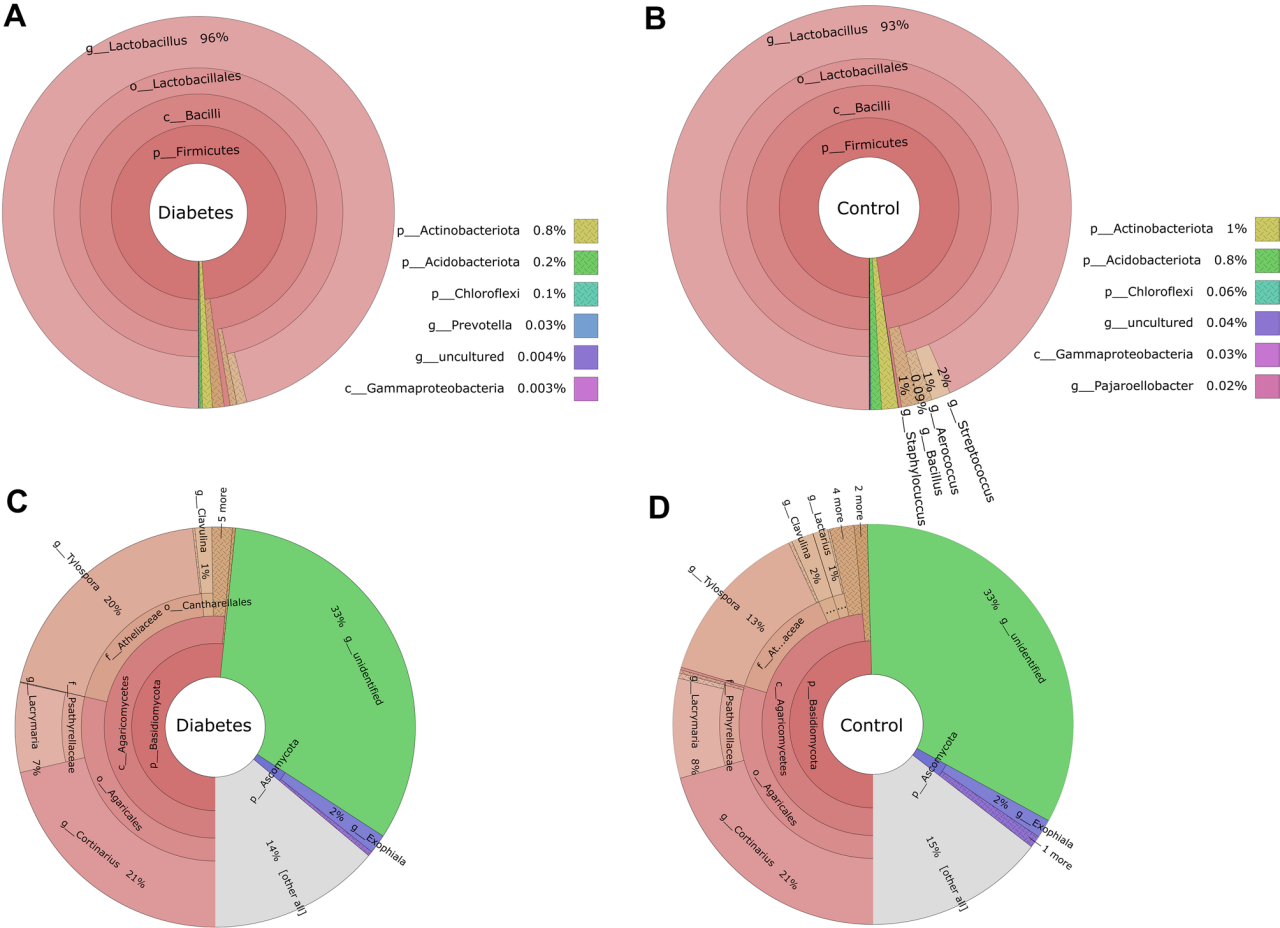
Averaged relative abundances of bacterial (**A**, **B**) and fungal (**C**, **D**) genera in Diabetes and Control groups

The total number of raw bacterial sequences was 1 526 318 and fungal sequences 1 095 814. The total frequency, after all quality filtering steps, of bacterial sequences steps was 435 993 (collapsed into 34 genera), 23 860 244 (290 pathways) predicted metabolic pathways, and 144 424 (collapsed into 25 genera) fungal sequences. The bacterial and predicted pathway data had 49 samples in the final analyses, while 44 samples remained in fungal data. The relative abundances of taxonomically assigned sequence reads for bacteria and fungi are presented in Fig. 1 and Supplementary Tables 1 and 2, respectively. The raw sequences were deposited to GenBank under the bioproject numbers PRJNA751475 for bacteria and PRJNA751714 for fungi.

**Fig. 2:**
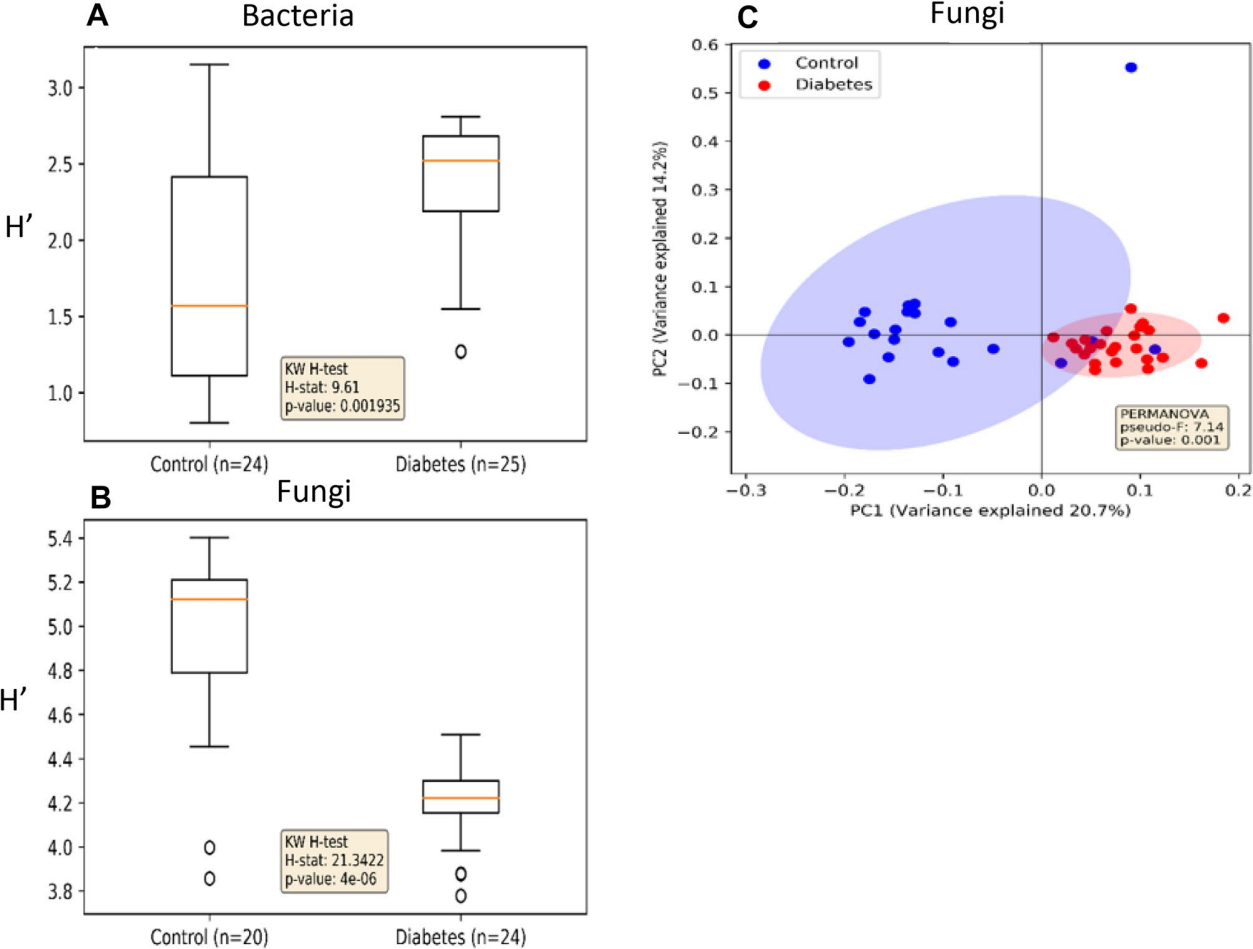
Alpha and beta diversity of bacteria and fungi in vaginal samples. Alpha diversity boxplots of Shannon’s diversity indices for Diabetes and Control group samples of **A** bacteria and **B** fungi. **C** Beta-diversity of fungal community in Diabetes and Control group samples

Alpha-diversity (Shannon, diversity within samples) of the vaginal bacteriome of Diabetes group mothers was higher compared to the Control group mothers (Fig. 2A). In contrast, the alpha-diversity of vaginal mycobiome was lower in the Diabetes group than in the Control group (Fig. 2B). Alpha-diversity of bacterial predicted pathways also tended to increase, but the result was not statistically significant (*P* = 0.08, Supplementary Fig. 1). We found a difference in beta-diversity (Bray–Curtis dissimilarity, diversity between samples) in the mycobiomes between Diabetes and Control groups (Fig. 2C). In the case of bacteriomes and predicted pathways, there were no statistically significant differences in beta-diversity (Supplementary Fig. 2).

**Fig. 3:**
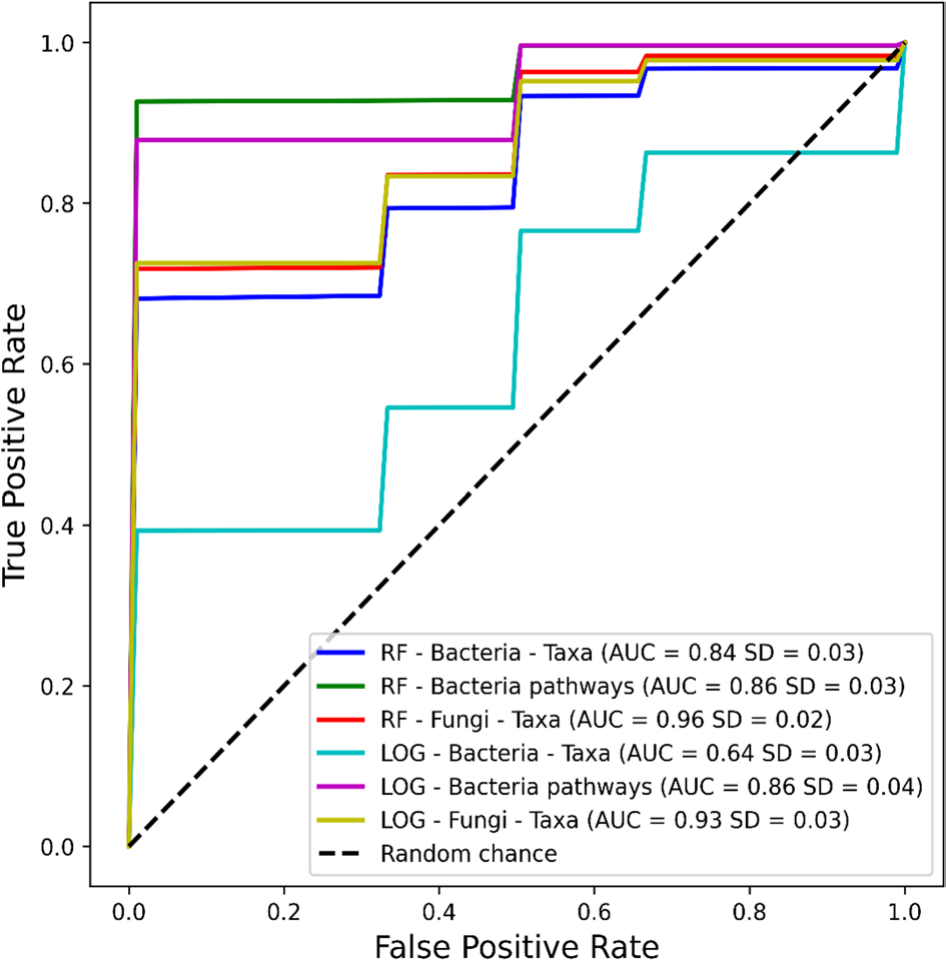
Cross-validated machine learning model performance when differentiating Diabetes and Control samples in the test samples. Dotted black line represents the performance of a model that is completely random (0.5 area under the curve, AUC), while a model that is always correct would have an AUC of 1.0

**Fig. 4:**
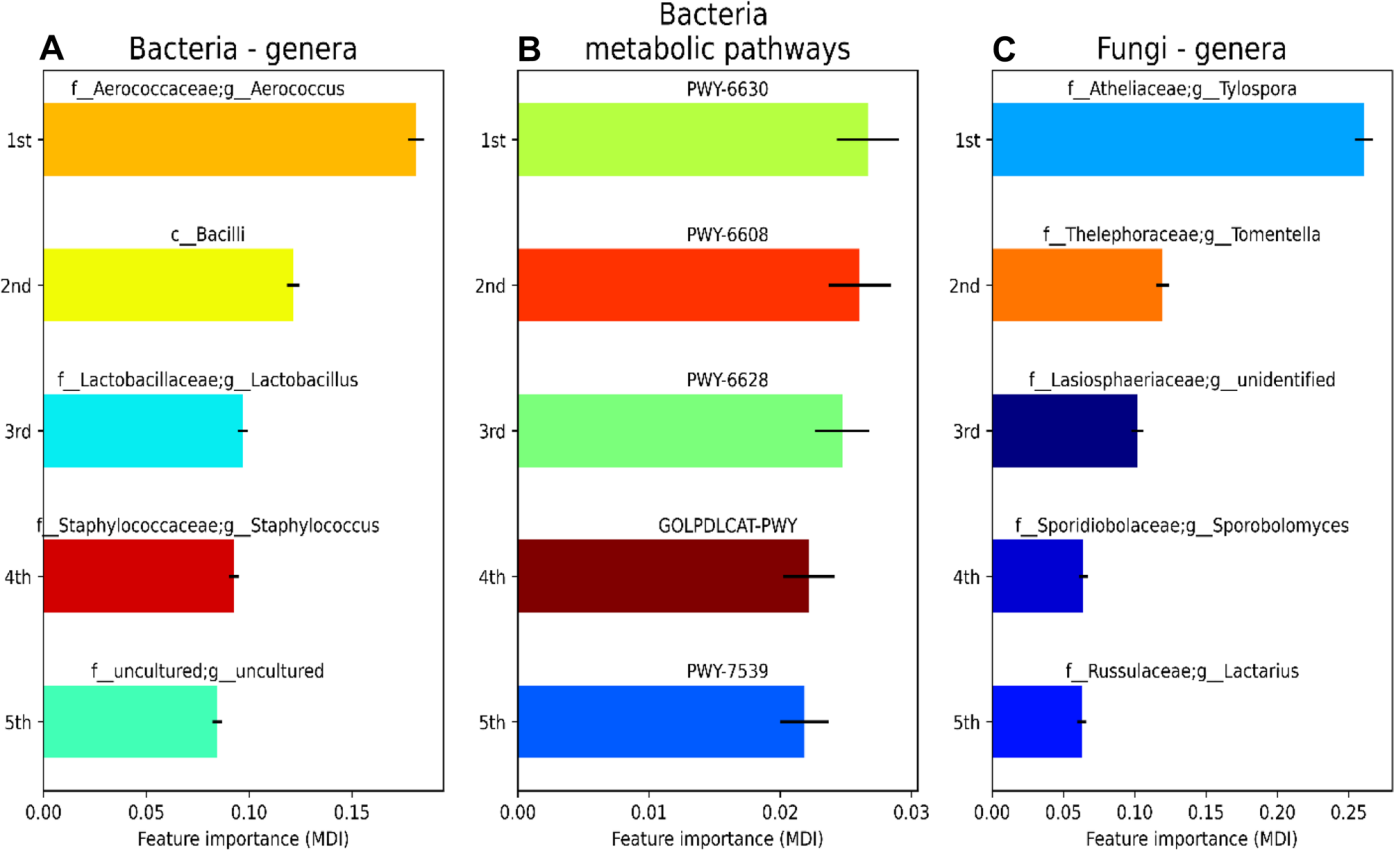
Importances of features used by random forest models (separate analyses for bacteria and fungi). Mean Decrease in Impurity (MDI) was used as the feature importance metric. Models were trained to predict unknown samples of Diabetes and Control groups in **A** bacteria, **B** pathways, and **C** fungi

Machine learning models differentiated well between Diabetes and Control group, where random forest (RF) models achieved high area under the curve (AUC) when using both bacteria (AUC = 0.86, SD = 0.03) and fungi (AUC = 0.93, SD = 0.02) (Fig. 3). Logistic regression (LOG) models had lower AUC values for all types of data, except in predicted metabolic pathway data derived from 16S sequences (0.86 AUC in both bacteria and fungi) (Fig. 3). Machine learning indicated several genera to be characteristic for Diabetes group both in separate (Fig. 4) and combined analyses (Supplementary Fig. 4) of bacterial and fungal genera. When bacteria and fungi data were combined for machine learning analyses, both RF (AUC = 0.96, SD = 0.02) and LOG (AUC = 0.93, SD = 0.04) models could predict with high accuracy between Diabetes and Control group test samples (Supplementary Fig. 3). Abundance data indicated several fungal genera and bacterial metabolic pathways to be associated with child T1D (Tables 1 and 2).

**Table 1:** Predicted metabolic pathway features in Diabetes and Control groups

Feature name	Diabetes vs Control	Effect
	Adjusted *p*-value	Direction
ASPASN-PWY	0.045	0.705
CENTFERM-PWY	0.049	0.577
HSERMETANA-PWY	0.031	0.498
PWY-5695	0.011	0.943
PWY-6507	0.033	0.635
PWY-6590	0.035	0.591
PWY-6608	0.004	1.058
PWY-6628	0.001	1.106
PWY-6630	0.001	1.097
PWY-6891	0.04	0.655
PWY-6892	0.04	0.685
PWY-6895	0.02	0.697
PWY-6901	0.024	0.612
RHAMCAT-PWY	0.036	0.592
THISYN-PWY	0.016	0.749

Effect: direction of change. Positive value: feature is more abundant in Diabetes group

**Table 2:** Aldex2 results for Fungi genera feature table

Feature name	Diabetes vs Control	Effect
	Adjusted *p*-value	Direction
K_Fungi;_;_;_;_;_	0.02	0.757
G_Exophiala	0.04	0.623
G_Cortinarius	0.006	0.889
G_Lacrymaria	0.026	0.593
G_Tylospora	0.001	1.194
G_Tomentella	0.044	– 0.69
G_unidentified	0.022	0.745

Effect = direction of change. Positive value = feature, i.e., fungal group is more abundant in Diabetes group

An amplified sequence variant (ASV) classified within the fungal genus *Tylospora* had the highest feature importances (MDI value) used in the random forest models to predict the differences between the mother groups (Fig. 4). The abundance of *Tylospora* sp. was also significantly higher in the Diabetes group samples (*P* = 0.001, Table 2, Supplementary Fig. 6, Supplementary Table 2). The bacterial genus *Aerococcus* was present at a higher frequency in Diabetes group samples than in Control group samples (96% vs 33% of samples, respectively) although an abundance of *Aerococcus* did not differ between groups (data not shown). Considering predicted metabolic pathways of bacteria, PWY-6630 (superpathway of L-tyrosine biosynthesis), PWY-6608 (guanosine nucleotides degradation III) and PWY-6628 (superpathway of L-phenylalanine biosynthesis) were the most important ones to predict the difference between Diabetes and Control groups (Fig. 4; Table 1).

## Discussion

We found differences in vaginal bacteriome and mycobiome between mothers who had a child with T1D (Diabetes group) and mothers with non-diabetic children (Control group). Alpha diversity of mother vaginal bacteriome was higher among the Diabetes group compared to the Control group. Increased alpha diversity, i.e., higher within-sample variation of the vaginal microbiome (bacteriome) of mother, related to the child T1D, has been previously reported by Tejesvi et al. [11]. In addition, observations of increased diversity in child’s bacterial gut microbiota in relation to maternal gestational diabetes have been observed by Wang et al. [12]. Accordingly, Vatanen et al. [31] reported increased diversity of child’s bacterial gut microbiota toward T1D onset in childhood. The relationship between increased diversity in mother’s vaginal bacteriome and child’s T1D suggests that there exist unidentified vaginal bacterial groups that play a role in development of T1D.

Tejesvi et al. [11] reported that within-genus beta-diversity of *Lactobacillus* was altered in the group of mothers with T1D children, and the bacterial genus *Prevotella* was characteristic to this group. High amounts of *Prevotella* have been found in vaginal microbiomes of women who have other than *Lactobacillus*-dominated microbiome, but *Prevotella* has also been linked to bacterial vaginosis [32]. In this study, *Prevotella* was absent from Control group samples and present in Diabetes group samples (at an average of 3% abundance), but this difference was not statistically significant. Drell et al. [15] found that increased microbiome diversity was linked to an increase in vaginal pH. In this study, we found a bacterial ASV classified as *Aerococcus* (Firmicutes, Bacilli) characterizing the difference between the Diabetes and Control group mothers. *Aerococcus*, which is a common member of air and vegetation microbial communities, is often found in the vaginal bacteriome, but it is also associated with bacterial vaginosis and urinary tract infections [33]. Unhealthy vaginal bacteriome of mother has been found to be linked to offspring health in a mouse model [34]. Whether changes in certain bacterial groups, such as in *Lactobacillus*, *Prevotella* or *Aerococcus* in mother vaginal microbiome are associated with child T1D, warrants further research.

We also predicted specific bacterial metabolic pathways in vaginal microbiomes that were associated with child T1D. Specifically, the predicted bacterial metabolic pathways characterizing differences between Diabetes and Control groups were related to amino acid biosynthesis and nucleotide degradation. The combination of these pathways may imply of direction of nitrogen usage within the bacterial communities, as nitrogen is specifically required for both amino acid and nucleotide synthesis. Because amino acids are building blocks for proteins, required for metabolic activity, and nucleotides are needed for replication of DNA and proliferation [35], the result suggests that bacteria were directing their nitrogen use toward survival and not cell division.

In contrast to the bacteriome, alpha-diversity of mycobiome was lower in Diabetes group mothers compared to the Control group mothers. Beta diversity of the mycobiomes also differed between the groups. Decreased diversity of vaginal mycobiome and altered community structure (beta-diversity) suggest that the vaginal mycobiome may play a more significant role in the full microbiome functions and, consequently, in the child’s gut microbiome, than has previously been thought. In general, recent findings have indicated that vaginal mycobiome is more diverse than previously thought [15, 16]. According to Hall & Noverr [36], the majority of vaginal fungi are opportunists, which suggests that they are sensitive to the living conditions of the vagina. Fungal communities of vaginally delivered children are also affected by environmental conditions, such as fungal dispersal via air, living environment, or caretakers [37]. In our study, the decreased diversity was related to several fungal genera/groups that were associated with vaginal mycobiome of mothers in the Diabetes group. In particular, *Tylospora* sp. was highly specific to the Diabetes group. Because the human mycobiomes are relatively little studied to date [15, 16], the importance of this finding remains to be discovered. This is due to the low capacity for taxonomic identification in the reference libraries, which can be expected to increase as more fungal reference samples of humans are identified [15]. However, regardless of poor classification of the taxa, identification of specific fungal groups in the mother vaginal mycobiome and changes in fungal diversity indices clearly indicate that fungal community changes may play a role in early onset of T1D. Dysbiosis, i.e., a change in the balance between gut bacteriome and mycobiome is reported to play a role in the onset of T1D in child gut microbiome [4]. We suggest that a similar kind of interaction between bacterial and fungal communities in mother’s vagina (as suggested also by Bradford & Ravel for vaginal communities in general [16]) may take place and that the altered microbiome is associated with the onset of T1D in a child [17].

Machine learning studies have shown that type 1 and type 2 diabetes can be predicted based on gut microbiome using both 16S and whole genome sequencing data [38–41]. In these studies, the machine learning models achieved moderate prediction performance in the range of 0.7–0.8 AUC, except for a deep learning-based model that achieved 0.9 AUC in a cohort of European women with type 2 diabetes [39]. In our study, the best models predicted T1D with a high performance of 0.86–0.96 AUC, indicating that the vaginal microbiome and mycobiome of the mother is a reliable predictor for T1D in children.

Our study has been carried out in one local population at maximum 11 years after the child delivery. Our findings therefore need to be corroborated by a longitudinal study, where the microbial sampling would be carried out at the time of birth and linked with a follow-up of child cohorts, similar to large-scale child diabetes studies (e.g., [42]), preferably covering several populations (countries and continents). In addition, our results rely on current microbial reference libraries, which in the case of humans, are changing fast (https://www.arb-silva.de, [43]). It is known that hormonal contraceptives may change the vaginal microbiota [44], and for this reason, the use of hormonal contraceptives was excluded in our study. None of the mothers had constant medication, or hormonal contraceptives, but we suggest that factors such as frequency of intercourse, glycemic index of food and the quality of hygiene products need to be taken into account in future studies. Vaginal microbiomes during childhood, reproductive-age and menopause are different also suggesting that within reproductive age vaginal microbiome is relatively stable [45, 46]. However, antibiotic use, gestation and mother hormonal status affect vaginal microbiome in short term [47, 48]. During the last trimester of pregnancy microbial diversity has been found to increase toward delivery [3].

Taken together, we have detected systematic variation in vaginal microbial communities between mothers with or without a child with diabetes in a one-population study. Although the current research has indicated that mother’s microbial communities may play a smaller role in determining the general microbiome of a neonate/child than have been previously assumed [3, 37], our results suggest that mother’s vaginal microbiome, even collected years after childbirth, may be linked with the development of T1D. This yet unknown link may act via vaginal hormonal status [49]. As our study was carried out in a relatively small geographical area, the results may be different in a wider population, and our observations should therefore be confirmed in a larger, cross-population study, where vaginal swab samples are preserved at the time of delivery.

## Supplementary Information

Below is the link to the electronic supplementary material.

Supplementary file1 (PDF 657 KB)

## Data Availability

Data is deposited in GenBank https://www.ncbi.nlm.nih.gov/genbank/ under the bioproject numbers PRJNA751475 for bacteria https://dataview.ncbi.nlm.nih.gov/object/PRJNA751475?reviewer=jia44284elai976f8b5otp063r and PRJNA751714 for fungi https://dataview.ncbi.nlm.nih.gov/object/PRJNA751714?reviewer=h1oaitfif938522cmfv3r5frq
