# Supplementary material for: Child type 1 diabetes associated with mother vaginal bacteriome and mycobiome
Source: Med Microbiol Immunol. 2022 Jun 14;211(4):185–94. doi: 10.1007/s00430-022-00741-w (PMC9304052; doi:10.1007/s00430-022-00741-w)
Supplement: Supplementary file 1 — Supplementary file1 (PDF 657 KB) [file 430_2022_741_MOESM1_ESM.pdf]

Child type 1 diabetes associated with mother vaginal bacteriome and mycobiome. Medical Microbiology and Immunology

Authors: Ruotsalainen AL*, Tejesvi MV, Vänni P, Suokas M, Tossavainen P, Pirttilä AM, Talvensaari-Mattila A, Nissi R

*corresponding author. **Affiliation:** Department of Ecology and Genetics, University of Oulu, Finland **E-mail:** annu.ruotsalainen@oulu.fi

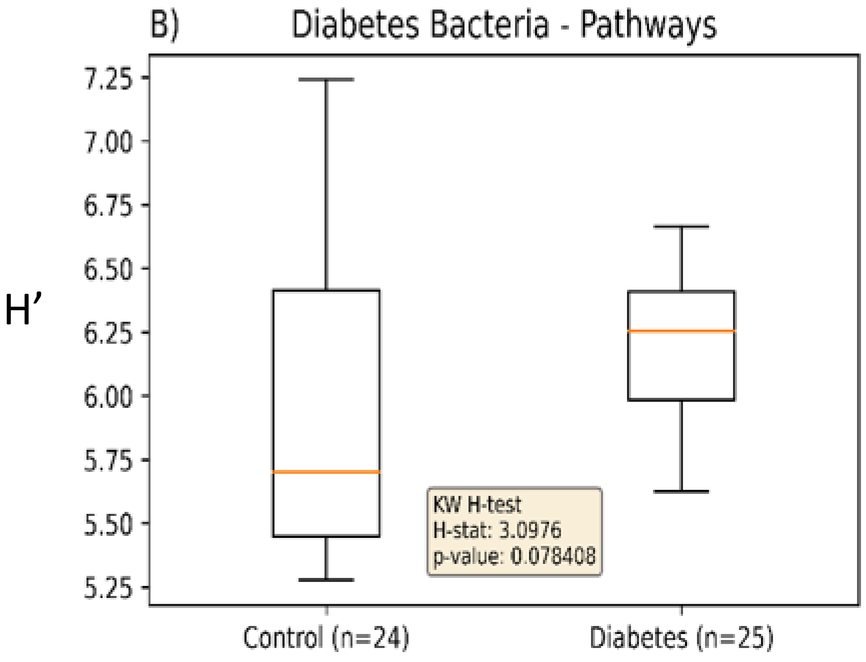

Supplementary Figure 1. Alpha diversity of metabolic pathways. Alpha diversity boxplots of Shannon's diversity indices for Diabetes and Control group samples.

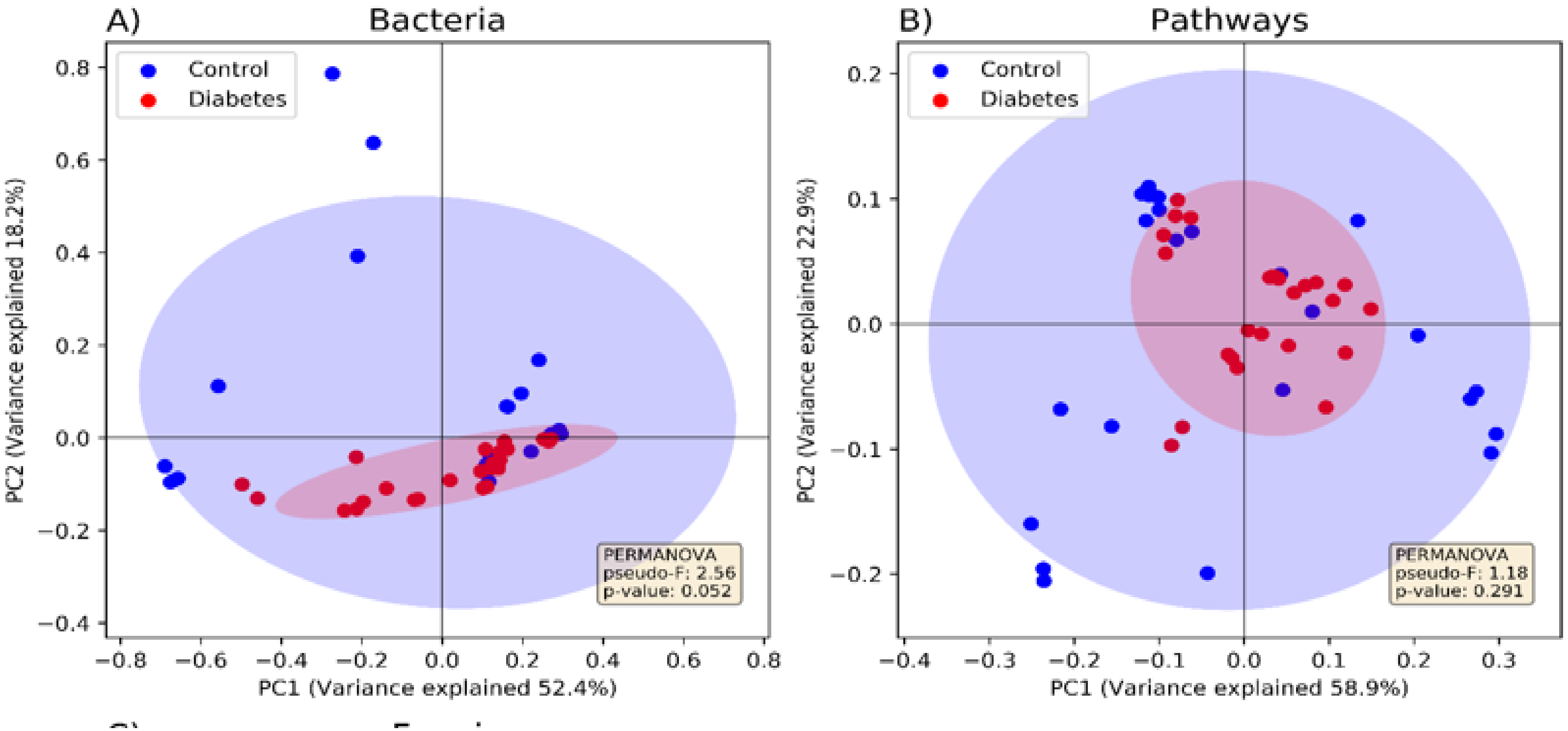

Supplementary Figure 2. Beta diversity analyses for A. bacteria and B. metabolic pathways.

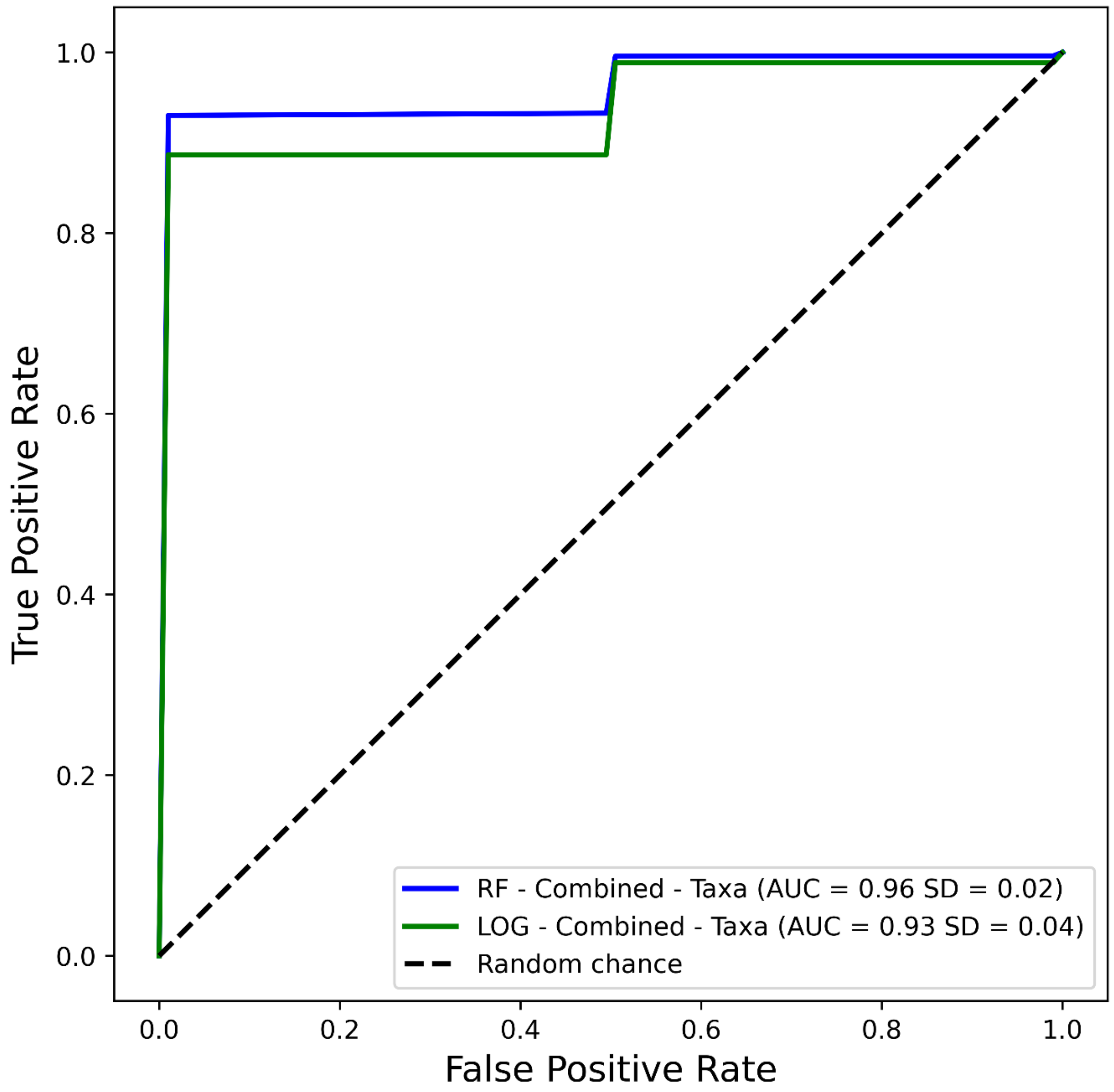

Supplementary Figure 3. Machine learning performances based on random forest (RF) and logistic regression (LOG) when predicting diabetes status of unseen samples of bacteria and fungi in combined analysis.

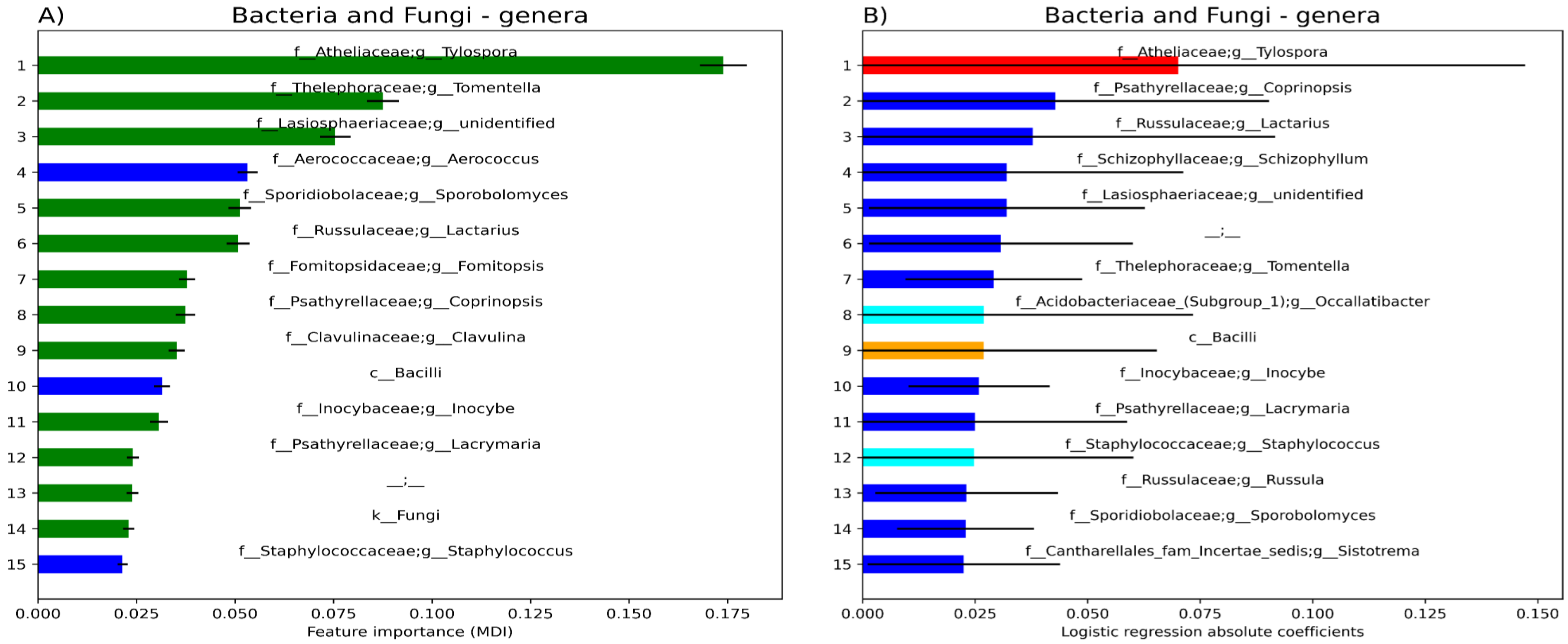

Supplementary Figure 4. Combined machine learning analyses based on random forest (A) and logistic regression (B). A: Random forest feature importances for combined genera models. Green features are fungi and blue are bacteria. **B:** Logistic regression model coefficients for combined genera models. Red: fungi important to diabetes, orange: bacteria important to diabetes, blue: fungi import to controls, and cyan: bacteria important to controls

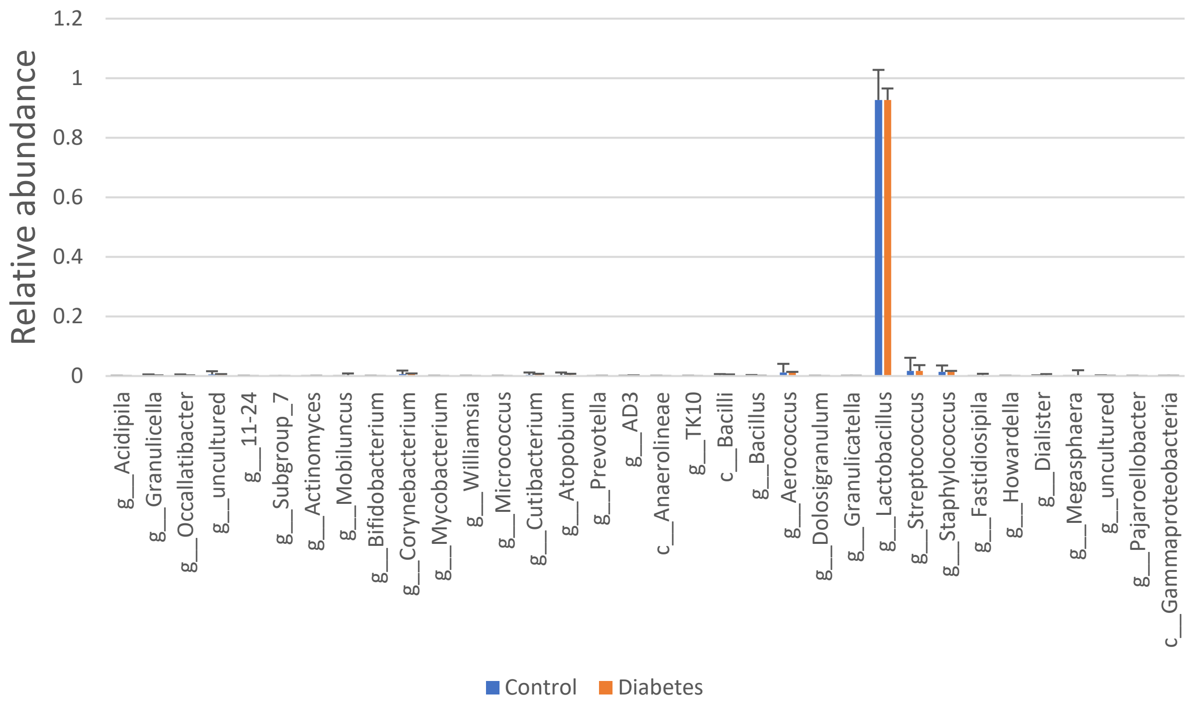

Supplementary Figure 5. Relative abundances (+SD) of bacterial taxonomic groups in Control and Diabetes groups.

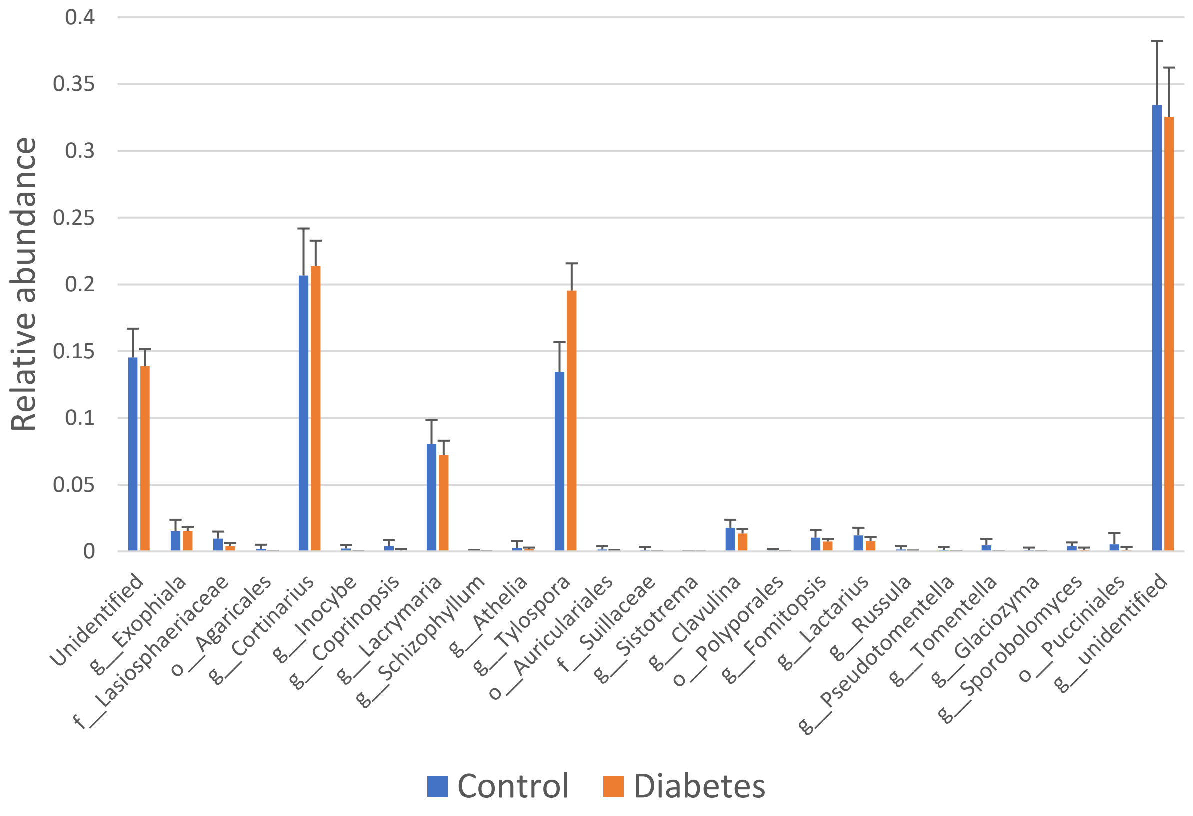

Supplementary Figure 6. Relative abundances (+SD) of fungal taxonomic groups in Control and Diabetes groups.

Supplementary Table 1. Relative abundance of bacterial taxa in the vaginal bacteriome in Control and Diabetes groups.

Control-mothers		Diabetes-mothers	
**Bacterial taxonomic group**	**%**	**Bacterial taxonomic group**	**%**
g__Lactobacillus	93.45 %	g__Lactobacillus	96.01 %
g__Streptococcus	1.52 %	g__Streptococcus	0.59 %
g__Staphylococcus	1.28 %	g__Megasphaera	0.55 %
g__Aerococcus	1.04 %	g__Staphylococcus	0.53 %
g__Corynebacterium	0.58 %	c__Bacilli	0.49 %
g__uncultured	0.51 %	g__Aerococcus	0.23 %
g__Cutibacterium	0.39 %	g__Corynebacterium	0.23 %
g__Atopobium	0.26 %	g__Cutibacterium	0.23 %
c__Bacilli	0.24 %	g__Dialister	0.20 %
g__Occallatibacter	0.15 %	g__Fastidiosipila	0.20 %
g__Granulicella	0.15 %	g__Mobiluncus	0.17 %
g__Bacillus	0.09 %	g__Atopobium	0.15 %
g__uncultured	0.04 %	g__uncultured	0.13 %
c__Gammaproteobacteria	0.03 %	g__AD3	0.10 %
g__Micrococcus	0.03 %	g__Occallatibacter	0.04 %
g__Dialister	0.03 %	g__Actinomyces	0.03 %
g__Granulicatella	0.02 %	g__Prevotella	0.03 %
g__TK10	0.02 %	g__Bacillus	0.02 %
g__Mycobacterium	0.02 %	g__Granulicella	0.01 %
c__Anaerolineae	0.02 %	g__Granulicatella	0.01 %
g__AD3	0.02 %	g__Bifidobacterium	0.01 %
g__Williamsia	0.02 %	g__Howardella	0.01 %
g__Pajaroellobacter	0.02 %	g__Williamsia	0.01 %
g__Dolosigranulum	0.02 %	g__uncultured	<0.01%
g__Bifidobacterium	0.02 %	g__Micrococcus	<0.01%
g__Acidipila	0.02 %	c__Gammaproteobacteria	<0.01%
g__11-24	0.01 %	g__Subgroup_7	<0.01%
g__Howardella	0.01 %	g__Dolosigranulum	<0.01%
g__Subgroup_7	0.01 %	c__Anaerolineae	<0.01%
g__Megasphaera	<0.01%	g__Acidipila	<0.01%
g__Actinomyces	NA	g__TK10	<0.01%
g__Mobiluncus	NA	g__11-24	<0.01%
g__Prevotella	NA	g__Mycobacterium	<0.01%
g__Fastidiosipila	NA	g__Pajaroellobacter	NA

Supplementary Table 2. Relative abundances of fungal taxa in the vaginal mycobiome in Control and Diabetes groups. g = genus, o = order, f = family.

Control-mothers		Diabetes-mothers	
**Fungal taxonomic group**	**%**	**Fungal taxonomic group**	**%**
o__Auriculariales	33.64 %	g__unidentified	32.54 %
g__Lactarius	20.44 %	g__Cortinarius	21.35 %
g__Sporobolomyces	14.92 %	g__Tylospora	19.55 %
g__Inocybe	13.62 %	Unidentified	13.89 %
g__Exophiala	8.07 %	g__Lacrymaria	7.22 %
o__Polyporales	1.68 %	g__Exophiala	1.55 %
g__Russula	1.53 %	g__Clavulina	1.35 %
g__Coprinopsis	1.12 %	g__Lactarius	0.79 %
o__Pucciniales	0.97 %	g__Fomitopsis	0.74 %
g__unidentified	0.95 %	f__Lasiosphaeriaceae	0.39 %
g__Fomitopsis	0.43 %	g__Athelia	0.18 %
g__Pseudotomentella	0.42 %	g__Sporobolomyces	0.14 %
g__Clavulina	0.42 %	o__Pucciniales	0.10 %
g__Glaciozyma	0.38 %	g__Coprinopsis	0.05 %
g__Sistotrema	0.29 %	o__Auriculariales	0.05 %
g__Cortinarius	0.22 %	g__Russula	0.03 %
o__Agaricales	0.20 %	g__Tomentella	0.02 %
Unidentified	0.15 %	g__Pseudotomentella	0.01 %
f__Lasiosphaeriaceae	0.13 %	o__Agaricales	0.01 %
g__Tylospora	0.11 %	o__Polyporales	0.01 %
g__Tomentella	0.09 %	g__Glaciozyma	0.01 %
g__Lacrymaria	0.09 %	g__Schizophyllum	0.01 %
g__Athelia	0.05 %	f__Suillaceae	<0.01%
f__Suillaceae	0.04 %	g__Inocybe	<0.01%
g__Schizophyllum	0.02 %	g__Sistotrema	NA
